# Prediction of pulmonary aspergillosis in patients with ventilator-associated pneumonia

**DOI:** 10.1186/s13613-023-01199-6

**Published:** 2023-11-07

**Authors:** Nicolas Massart, Emma Plainfosse, Yanis Benameur, Clarisse Dupin, Florence Legall, Anne Cady, Frederic Gourmelin, François Legay, Nicolas Barbarot, Eric Magalhaes, Pierre Fillatre, Aurélien Frerou, Florian Reizine

**Affiliations:** 1 Service de Réanimation, CH de St BRIEUC, 10, Rue Marcel Proust, 22000 Saint-Brieuc, France; 2 Service d’Anesthésie et de Réanimation Chirurgicale, CHU de Rennes, 2, rue Henry le Guilloux, 35000 Rennes, France; 3 Service de Réanimation, CH de QUIMPER, 14Bis Avenue Yves Thépot, 29107 Quimper, France; 4 Service de Microbiologie, CH de St BRIEUC, 10, rue marcel Proust, 22000 Saint-Brieuc, France; 5 Service de Microbiologie, CH de QUIMPER, 14bis Avenue Yves Thépot, 29107 Quimper, France; 6 Laboratoire de Biologie Médicale, CH de Vannes, 20, bd Maurice Guillaudot, 56000 Vannes, France; 7 Service de Réanimation, CH de Saint-Malo, 1 rue de la marne, 35400 Saint-Malo, France; 8 Service de Réanimation, CH de Vannes, 20, bd Maurice Guillaudot, 56000 Vannes, France

**Keywords:** Critical care, Pneumonia, Acquired infection, Pulmonary aspergillosis, Prediction score

## Abstract

**Background:**

Predictors of ICU-acquired pulmonary aspergillosis (IPA) are not well-established in critically ill patients with ventilator-associated pneumonia (VAP), making IPA commonly misdiagnosed and anti-fungal therapy delayed. We aimed to develop a clinical score for prediction of IPA among patients with VAP.

**Methods:**

Mechanically ventilated patients who developed VAP in 4 ICUs in Bretagne, Western France, were included. The score was constructed in a learning cohort, based on predictors of IPA in logistic regression model, and validated in a validation cohort.

**Results:**

Among 1636 mechanically ventilated patients, 215 developed VAP but only 39 developed IPA (4 possible and 35 probable/putative) (18%). Most cases (31/39) were documented through a positive broncho-alveolar sample culture. Independent predictors of IPA were immunodepression (including onco-hematological disorder, immunomodulatory treatment, solid organ transplant, neutropenia < 0.5G/L and high-dose steroids ≥ 1 mg/kg/day of prednisolone equivalent) (*p* = 0.001; score = 1 point) and lymphocyte count at admission < 0.8 G/L (*p* = 0.019; score = 1 point). Operational values of the predictive score in the learning/validation cohort were 50%/52% sensitivity and 90%/87% specificity, respectively, for high PiPa score (score = 2) and 94%/91% sensitivity and 44%/46% specificity, respectively, for moderate PiPa score (score = 1). Finally, the AUC for the prediction of IPA was 0.783 in the learning cohort and 0.770 in the validation cohort.

**Conclusions:**

We evaluated a clinical score with good predictive value which may help to predict IPA in patient with VAP. External validation will be needed to confirm our preliminary findings.

**Supplementary Information:**

The online version contains supplementary material available at 10.1186/s13613-023-01199-6.

## Background

Fungi are the deadliest but paradoxically the most disregarded agent of intensive care unit (ICU) acquired infection [1–3]. Once thought to occur mostly in immunocompromised patients, ICU-acquired pulmonary aspergillosis (IPA) has lately been reported with high incidence among critically ill patients [1–8]. Recently, Loughlin et al. reported up to 12.4% of IPA in non-specific population of ICU patients with suspicion of ventilator-associated pneumonia (VAP), but this incidence rate might be higher in targeted settings [1–8]. Notably, IPA incidence might be lower in patients with recognized risk factors such as hematological malignancies than in other uncommonly described populations [5, 9]. Beyond the immunological status, the condition of the patient requiring intensive care and the subsequent immunomodulation may favor the development of IPA. The unrecognized burden of IPA may promote delayed treatment and increased mortality [2, 5, 8, 10, 11] making early mycological screening crucial for patients most at risk of developing such a condition. Since identifying patients at higher risk of IPA may help physicians in clinical diagnosis and treatment, we aimed to develop a clinical score for prediction of IPA among patients with suspected VAP.

## Methods

### Setting and patients

We performed a secondary analysis of a multicenter prospective observational study [6, 12] conducted in western France. In the present study, mechanically ventilated patients admitted in four participating ICUs from January 1st 2020 until December 31^th^ 2022 were eligible but only those who developed VAP were finally included. Patients deprived of freedom by a judicial or administrative decision, pregnant women and patients younger than 18 years of age were excluded. The study protocol received approval from the Rennes Hospital ethics committee (Comité d’Éthique du CHU de Rennes, avis 19-52). Patients or closest relative were informed of the anonymous prospective collection of data and could refuse to participate in the study. In case of refusal, data were not collected. This manuscript follows the STROBE statement for reporting cohort studies.

Strategies for ICU-acquired infection prevention in participating ICUs are available in Additional file 1.

### Definition

Infection was considered acquired in the ICU when diagnosed at least 48 h after admission and incubation was not on-going upon admission. VAP was considered as acquired in the ICU only in patients without microbiological or suggestive radiological findings in the first 48 h of stay in whom diagnosis was confirmed after at least 48 h of mechanical ventilation. VAP diagnosis relied on clinical signs (fever, purulent sputum, hypoxia), radiological findings (new infiltrate on chest X-ray or CT scan), and leukocytosis (> 10 G/L) in patients intubated for more than 48 h [13]. In patients with ARDS, for whom it is difficult to demonstrate radiological deterioration, this criterion has been removed from the definition of VAP [14, 15]. Respiratory samples for VAP diagnosis were performed either using fiberoptic broncho-alveolar lavage or endotracheal aspiration, according to local practices. Thresholds for lung sample positivity were established at 10^4^ colony forming units (cfu)/mL for BAL and 10^6^ cfu/mL for tracheal aspirate [16]. Each center had a nosocomial infection committee for the prevention and the prospective census of acquired infections and applied the recommendations of the French Society for Hospital Hygiene for the prevention and treatment of infection.

For the purpose of the study, only the first VAP episode was evaluated.

COVID-19 and influenza were diagnosed with a positive RT-PCR from a respiratory tract sample. As part of local protocol, all patients requiring intubation had a pulmonary sample taken for microbiological testing early after admission.

Immunodepression was considered in patients with either onco-hematological disorder, immunomodulatory treatment, solid organ transplant, neutropenia < 0.5 G/L and high-dose steroids > 1 mg/kg/day of prednisolone equivalent (regardless of treatment duration). When immunodepression was not present at admission, it was only considered when the factor causing immunodepression occurred before first VAP, whereas in other cases (i.e., steroids after VAP for example) patients were considered as immunocompetent.

### Method for aspergillosis diagnosis

Notably, all respiratory samples from all the participating ICUs were sent for bacteriological analysis and also cultured in a dedicated fungal media for aspergillosis screening. This aspergillosis screening was conducted systematically, whenever patients were immunocompromised or not or had COVID-19 or not. Fungal culture was performed in respiratory samples in Sabouraud-chloramphenicol media and species identification was by MALDI-ToF mass spectrometry. Molecular detection was also performed on respiratory samples after DNA extraction with a qPCR assay targeting the mitochondrial gene and the 28S rDNA region. Galactomannan was measured in serum with an index cut-off of 0.5 and in BAL, with an index cut-off of 1.0, but were not measured in endotracheal aspiration. Chest computed tomographies were analyzed by a senior radiologist and was considered compatible with acquired pulmonary aspergillosis when lesions were not present at admission but developed during ICU stay [4].

Patients were classified as having possible, putative, probable or proven aspergillosis according to the modified AspICU, Influenza associated pulmonary aspergillosis (IAPA) and COVID-19 associated pulmonary aspergillosis (CAPA) criteria when indicated [17–20]. Algorithm used for IPA diagnosis is detailed in Additional file 2: Fig. S1.

### Primary and secondary endpoints

Primary aim of the study was to construct and validate a bedside applicable score to predict IPA in patients with suspicion of VAP. Secondary aims were to describe IPA incidence, specific risk factors and outcomes.

Then, predictors of IPA were analyzed in a subset of patients with pre-existing immunodepression. These patients are known to be at higher risk of IPA and pre-test likelihood can modify score performance. Finally, predictors of VAP were analyzed in the subset of patients in whom VAP diagnosis included a BAL.

### Score construction

For the purpose of score construction and validation, the study population has been separated into two cohorts. Using computer randomization (software Excel 2016, function “alea”), each patient was randomly selected to constitute either the learning cohort or the validation cohort.

**Fig. 1:**
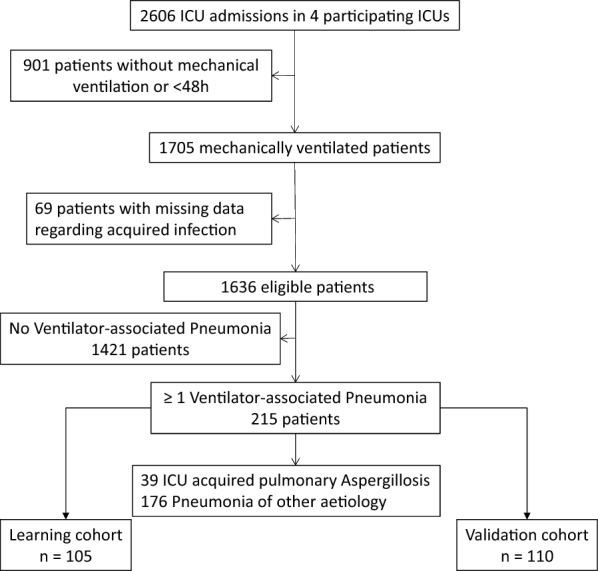
Clinical algorithm for VAP and IPA diagnosis

The score was constructed using the dataset of patients of the learning cohort. (i) Risk factors were identified using a uni- and multi-variable logistic regression model. Variables known before or at the time of VAP diagnosis were included in multivariable analysis when *p*-value was < 0.2 in the univariate analysis. Then, a reduced model was determined using backward selection procedures with Akaike’s information criterion as the stopping rule. (ii) Variables identified as predictor of IPA in the multivariable regression model were retained for score construction. The regression coefficient of each variable was divided by the smallest coefficient to obtain the weight of the variable in the definite score. (iii) For each patient, the score was calculated as the sum of points attributed to each predictor. Patients with lower scores were expected to have a low risk of IPA, whereas those with higher scores were expected to be at high risk. (iv) Discrimination was evaluated using the area under the ROC curve (AUC). (v) Finally, predictive values of the score were confirmed in the validation cohort (Fig. 1).

Full statistical analysis section is available in Additional file 1.

## Results

**Fig. 2:**
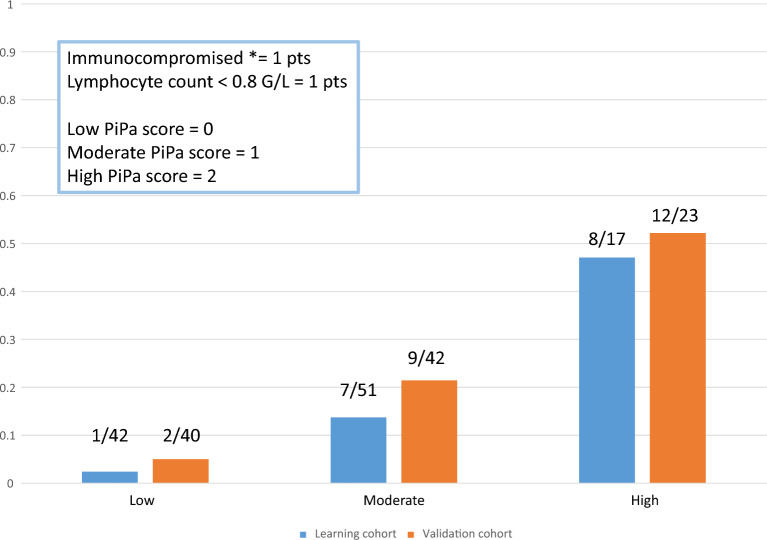
Flowchart

Among 1636 eligible patients, 215 developed VAP during ICU stay and were included in the study (Fig. 2). Among those 215 patients, mean age was 65 years [55–71], 200 (93%) were admitted for medical reasons, 68 (32%) were considered as immunocompromised, 106 (49%) received early steroids of whom 22 received > 1 mg/kg/day of prednisolone equivalent. Time from admission to VAP onset was 9 days [5–13].

There were 259 micro-organisms identified in the 215 VAP episodes analyzed, of which 79 were *Enterobacteriaceae*, 56 were *Staphylococcus aureus*, 41 were non-fermenting Gram-negative *Bacilli* and 23 were *Streptococci*, 73/215 VAP (33%) were poly-microbial.

Thirty-nine patients (18%) were diagnosed with IPA, of whom 4/39 had possible IPA and 35/39 probable/putative IPA. Most cases (31/39) had a broncho-alveolar lavage culture positive for Aspergillus, mainly *Aspergillus fumigatus* (29/31). Of note, 13/39 had both positive BAL culture and Galactomannan, 15/39 only had positive BAL culture only, 3/39 had positive galactomannan only and 3/39 only had positive PCR. Bacteria was co-isolated in 17/39 IPA cases, mainly constituted of *Enterobacteriaceae* (14/17) and non-fermenting Gram-negative bacilli (3/17).

Patients with IPA were older (70 years [63–73] vs 64 years [53–70] *p* = 0.003), were more frequently considered as immunocompromised (22/39 (56%) vs 37/176 (21%) *p* < 0.001), received more frequently high-dose steroids early after admission (13/39 (33%) vs 9/176 (5.1%) *p* < 0.001), had lower lymphocyte count (0.54 G/L [0.24–0.74] vs 0.79 [0.55–1.28] *p* < 0.001) and a lower PaO2/FiO2 ratio (111 mmHg [86–129] vs 137 mmHg [105–209] *p* = 0.002) at admission (Table 1; Additional file 1: Table S1). At VAP onset, patients with IPA had a lower body temperature (38.5 °C [38.0–38.6] vs 38.7 °C [38.1–39.0], *p* = 0.046) and were less likely to have purulent sputum (19/39 (48.7%) vs 136/176 (77.3%) *p* < 0.001) (Table 2). BAL was performed in 123 patients (57%) and proportion of patients with BAL (vs ETA) for VAP diagnosis was similar in between with or without COVID-19 (58% vs 56%, respectively, *p* = 0.84). Conversely, immunocompromised patients had more frequently BAL than immunocompetent patients (71% vs 51%, respectively, *p* = 0.011).

**Table 1:** Baseline characteristics and outcomes in full population

Variables	Non aspergillus related VAP	ICU-acquired pulmonary aspergillosis	*p*-value
	*n* = 176	*n* = 39	
Age, year	64 [53–70]	70 [63–73]	0.003
Male—no. (%)	136 (77.3)	33 (84.6)	0.426
Year of admission			0.687
2020—no. (%)	65 (36.9)	12 (30.8)	
2021—no. (%)	79 (44.9)	18 (46.2)	
2022—no. (%)	32 (18.2)	9 (23.1)	
*Immunocompromised—no. (%)*	44 (25.0)	24 (61.5)	< 0.001
Oncohematological disorder—no. (%)	26 (14.8)	12 (30.8)	0.034
Immunomodulatory treatment—no. (%)^a^	9 (5.6)	14 (41.2)	< 0.001
Solid organ transplant—no. (%)	3 (1.7)	3 (7.7)	0.075
Neutropenia < 0.5 G/L	5 (2.8)	7 (17.9)	0.001
Steroids	77 (43.8)	29 (74.4)	0.001
< 1 mg/kg of prednisolone equivalent	69 (41.3)	17 (63.0)	0.059
≥ 1 mg/kg of prednisolone equivalent	9 (5.1)	13 (33.3)	< 0.001
Simplified Acute Physiology Score II	50 [37–68]	47 [36–52]	0.149
Reason for admission			
Medical (vs surgical)—no. (%)	161 (91.5)	39 (100.0)	0.123
Trauma—no. (%)	12 (6.8)	0 (0.0)	0.130
COVID-19—no. (%)	97 (55.1)	25 (64.1)	0.397
Influenza—no. (%)	1 (0.7)	2 (6.9)	0.077
Period of admission			0.660
Winter—no. (%)	54 (30.7)	12 (30.8)	
Spring—no. (%)	52 (29.5)	8 (20.5)	
Summer—no. (%)	24 (13.6)	7 (17.9)	
Fall—no. (%)	46 (26.1)	12 (30.8)	
Localization before admission			0.078
Other ICU—no. (%)	29 (16.5)	1 (2.6)	
Home—no. (%)	82 (46.6)	21 (53.8)	
Long care facility—no. (%)	4 (2.3)	0 (0.0)	
Acute care ward—no. (%)	61 (34.7)	17 (43.6)	
Early management			
Systemic antibiotic at admission—no. (%)	115 (65.3)	30 (76.9)	0.227
Vascular catheter—no. (%)	169 (96.0)	39 (100.0)	0.443
Multiple site decontamination—no. (%)	45 (25.6)	14 (35.9)	0.234
Biological parameters at admission			
Creatininemia, µmol/L	85 [64–135]	84.50 [62–139.50]	0.660
White blood cell count, Giga/L	10.95 [8.83–16.43]	8.67 [6.20–11.78]	0.004
Neutrophilic count at admission, Giga/L	9.20 [6.37–14.56]	7.15 [3.55–10.36]	0.002
Lymphocyte, Giga/L	0.79 [0.55–1.28]	0.54 [0.24–0.74]	< 0.001
PaO_2_/FiO_2_, mmHg	137 [102–209]	111 [86–129]	0.002
Outcomes			
In ICU death—no. (%)	52 (29.5)	21 (53.8)	0.007
Length of stay, days	26 [17–39.50]	27 [16.50–38.50]	0.907
Length of mechanical ventilation, days	22 [13–36]	21 [11.50–34.50]	0.319

^a^Immunomodulatory treatments included chemotherapy for solid organ tumor in 4 cases, for hematological malignancy in 7 patients, 4 patients received tacrolimus for solid organ transplant, 3 received tocilizumab for COVID-19, 1 patient received rituximab for acute vasculitis, 1 received methotrexate and treatment was not known by the investigator for 6 patients (some patients may have received multiple immunomodulatory treatments)

**Table 2:** Characteristics at ventilator-associated pneumonia onset in full population (learning and validation cohorts)

Variables	Bacterial VAP	Acquired pulmonary aspergillosis	*p*-value
	*n* = 176	*n* = 39	
Data regarding VAP			
Time from admission to VAP, days	8 [5–12]	10 [4–14]	0.599
Purulent sputum at onset—no. (%)	136 (77.3)	19 (48.7)	< 0.001
Body temperature at onset, °C	38.70 [38.10–39]	38.50 [38–38.60]	0.046
White blood cell count at onset, G/L	12 [9.91–16.50]	10.36 [7.78–13.25]	0.014
PaO_2_/FiO_2_ at onset, mmHg	180 [132.50–241.50]	175 [125–221.14]	0.825
Septic shock at onset—no. (%)	53 (34.0)	9 (25.7)	0.457
New pulmonary infiltrate at onset—no. (%)	67 (38.0)	18 (46.1)	0.350
Polymicrobial VAP—no. (%)	56 (31.8)	17 (43.6)	0.223

*SC* standard care, *MSD* multiple site decontamination, *ICU* intensive care unit, *COVID-19* SARS-COV 2 associated infection disease, *VAP* ventilator-associated pneumonia

### Score construction

Whole population was randomly split into a learning (*n* = 110) and a validation cohort (*n* = 105). There were no statistical differences in baseline characteristics between patients of the learning and the validation cohorts (Additional file 1: Table S2). Predictors of IPA were evaluated in the learning cohort, using a uni-variable (Additional file 1: Table S3) and a multivariable logistic regression model with stepwise backward elimination using Akaike criteria as a stopping rule. In the final model, immunodepression (including onco-hematological disorder, immunomodulatory treatment, solid organ transplant, neutropenia < 0.5 G/L and high-dose steroids ≥ 1 mg/kg of prednisolone equivalent) (OR = 7.19; 95%CI [2.16–23.98] *p* = 0.001) and lymphocyte count at admission < 0.8 G/L (OR = 4.56; 95%CI [1.27–16.32] *p* = 0.019) were independently associated with a higher risk of IPA (Table 3). Based on this model, the weight of each variable was immunodepression = 1 point (regression coefficient = 1.97), lymphocyte count at admission < 0.8 G/L = 1 point (regression coefficient = 1.52). There were 1/42 IPA among patients with low PiPa score (score 0), 7/51 IPA in patients with moderate PiPa score (score = 1) and 8/17 IPA in patients with high PiPa score (score = 2) (Fig. 2; Additional file 2: Fig. S1). Operational values of the predictive score are available in Table 4, for high PiPa score, sensitivity was 50% and specificity 90%, conversely, for moderate or high score (PiPa score ≥ 1), sensitivity was high, at 94% but specificity was only 44% (Table 4). Finally, the AUC for the prediction of IPA was 0.783 (Additional file 1: Fig. S2).

**Table 3:** Predictor of ICU-acquired pulmonary aspergillosis in the learning cohort (multivariable logistic regression with stepwise backward elimination using Akaike criteria as stopping rule)

Variables	OR	95%CI	*p*-value	*Β*-estimate	Score for score calculation
Immunodepression^a^	7.19	2.16–23.98	0.001	1.97	1
Lymphocyte count at admission < 0.8 G/L	4.56	1.27–16.32	0.019	1.52	1

^a^Including onco-hematological disorder, immunomodulatory treatment, solid organ transplant, neutropenia < 0.5 G/L and high-dose steroids > 1 mg/kg/day of prednisolone equivalent

**Table 4:** Operational values of the PiPa score

Grade	Sensitivity	Specificity	Predictive positive value	Negative predictive value	
Low PiPa score = 0 (%)	–/–	–/–	–/–	–/–	
Moderate or high PiPa score ≥ 1 (%)	94/91	44/46	22/32	98/95	
High PiPa score = 2 (%)	50/52	90/87	47/52	91/87	

Values are displayed for the learning/validation cohort, respectively

### Score validation

Validation cohort constituted of 105 patients of whom 23 had IPA. In this cohort, there were 2/40 IPA in patients at low risk as compared with 9/42 IAP in patients with moderate risk and 12/23 IPA in patients with high score. Operational values of the predictive score in the validation cohort was 52% sensitivity and 87% specificity for high PiPa score, and 91% sensitivity and 46% specificity for moderate or high PiPa score (PiPa score ≥ 1) (Table 4). AUC was 0.770 (Additional file 1: Fig. S2).

### Risk factor for IPA in patients sub-groups

BAL is a cornerstone of IPA diagnosis therefore a sensitive analysis was conducted in patients in whom VAP diagnosis included a pulmonary sample performed through a BAL (*n* = 123). In this subgroup, immunodepression (OR = 3.90; 95%CI [1.53–9.99] *p* = 0.004) and lymphocyte count at admission < 0.8 G/L (OR = 9.03; 95%CI [3.24–25.15] *p* < 0.001) were independently associated with a higher risk of IPA. Notably, patients with lymphocyte count at admission < 0.8 G/L had the same proportion of BAL for VAP diagnosis than patients with higher lymphocyte count (*n* = 60 (57%) vs *n* = 63 (57%) *p* = 1). In addition, the proportion of patients with BAL (vs ETA) for VAP diagnosis was similar between patients with or without COVID-19 (58% vs 56%, respectively, *p* = 0.84), conversely, immunocompromised patients had more frequently BAL than immunocompetent patients (*n* = 48 (71%) vs *n* = 75 (51%) *p* = 0.011).

Finally, a post hoc analysis of risk factors of IPA in full population is also available in Additional file 1: Table S3 while analysis of risk factors for IPA in immunocompromised and immunocompetent patients is available in Additional file 1: Table S4. Since patients with pre-existing immunodepression are known to be at higher risk of IPA, predictors were specifically analyzed in this setting. There were 136 patients with pre-existing immunodepression (including low-dose steroids). In this subgroup, there were 28/136 (21%) IPA. In a multivariable logistic regression with stepwise backward elimination using Akaike criteria as a stopping rule, high-dose steroids (OR = 6.70, 95%CI [1.49–30.08] *p* = 0.013) and immunomodulatory treatment before admission (OR = 18.2, 95%CI [3.63–91.60] *p* < 0.001) were independently associated with IPA.

Three patients with low PiPa score developed IPA. First case developed in a 28-year-old patient admitted for varicella zona zoster associated pneumonia (case 22 in Additional file 1: Table S1). The patient received voriconazole and was discharged alive.

The two last cases developed in two patients, 62 and 56 years old, respectively, who were immunocompetent COVID-19 patients who received early steroids (dexamethasone 6 mg daily) (case 36 and case 37 in Additional file 1: Table S1). Both received voriconazole but last patient died early after diagnosis.

### Outcome

Length of stay in the ICU and in the hospital did not differ between patients with or without IPA (Table 1). However, 21/39 (54%) patients with IPA died in the ICU, as compared with 52/176 (30%) patients without IPA (*p* = 0.007) (Additional file 1: Fig. S3). This difference remained after adjustment on SAPS II, lymphocyte count at admission and immunodepression (HR = 2.17; 95%CI [1.23–3.82] *p* = 0.007).

## Discussion

In this observational study that evaluated 1636 mechanically ventilated ICU patients, 215 had VAP, but only 39 cases of IPA were observed. Using easily available variables, we constructed and validated an easy-to-use score for prediction of IPA in patients with VAP, with good predictive capacity. Patients considered at low risk (PiPa score = 0) have low IPA incidence < 5%, whereas those at high risk (PiPa score score = 2) have a high incidence > 45%.

Strikingly, in the present work that evaluates a large unselected population of 1636 patients at risk of IPA accounting for 16 949 patients’ days only 39 cases (2.3/1000 patients-days) were reported, which is very low. In other studies that do not focus on patients with VAP, higher IPA incidence has been reported, around 7% in patients with VV-ECMO support, 15% in non-COVID-19 related ARDS, 28% in cirrhotic patients and higher than 19% in influenza [3, 8, 21, 22]. Even if delay between intubation and IPA onset varies greatly from one study to another, most of the cases are diagnosed after 48 h of stay suggesting that they were ICU acquired. However, we cannot rule out that pulmonary aspergillosis was present on admission. Only a prospective observational study with systematic measurement of plasmatic and BAL galactomannan, associated with BAL dedicated culture will allow to properly evaluate IPA incidence in patients with VAP. Concerning patients with VAP, Loughlin et al. retrospectively analyzed a cohort of 194 mechanically ventilated ICU patients with VAP suspicion, all of whom had BAL fluid culture and galactomannan measurement (BAL and serum) [1]. They reported an IPA incidence around 12.4%, closer to the 18% observed in our study. They highlighted for the first time an unsuspected high rate of IPA in patients with suspected VAP. Strikingly, in our study either this fungus was frequently present, in the same proportion than non-fermenting Gram-negative bacilli an extensively studied agent of VAP. The question that arises from the study of Loughlin et al. was whether systematic screening for IPA should be performed in all patients with suspected VAP [23]. Unfortunately, their study was not powered to identify specific risk factors for IPA in an unselected population. In the present work, we constructed and validated a score that can help physicians in decision-making about when to screen for IPA and eventually when to consider empirical therapy. Patients with low PiPa scores can be ruled out for IPA diagnosis. However, in patients with high PiPa scores, physicians should be aware of the high proportion of IPA, close to 50%, and IPA should be considered.

Physiopathology of IPA is not consensual. Regarding host factors, it is well recognized that critical illness, especially sepsis, promotes immune disorders that may favor secondary-infection acquisition [24, 25]. However, regarding fungus, it is not clear how aspergillus contaminates the respiratory tract. Interestingly, when Contou et al. studied 423 ARDS patients, they noticed 8.3% of patients with at least one respiratory tract sample positive for Aspergillus early after admission, of whom only half were diagnosed with IPA [26]. We may hypothesize that patients may acquire aspergillosis colonization before intubation but only develop IPA in unfavorable conditions.

Predictors of IPA in our study are not surprising. Immunodepression has been largely documented as risk factors of IPA [2, 26, 27]. While susceptibility to invasive pulmonary aspergillosis has been mainly linked to myeloid cell dysfunction with neutropenic patients being particularly at risk for these infections, in the present study, lymphopenia was found to be a good predictor for IPA. Notably, beyond myeloid impairment, alterations of adaptive immune compartments, in particular T cell exhaustion, are also associated with severe fungal infections and PD-1 blockade in a murine model of post-sepsis aspergillosis reinvigorates T cells and attenuates secondary aspergillosis [28]. In addition, some authors have reported beneficial effects of immune checkpoint inhibitors combined with interferon-gamma in patients with invasive fungal diseases [29, 30]. These data suggest that abnormalities of the lymphoid compartments may also significantly affect anti-Aspergillus response. Some authors support that pro-inflammatory immune response in sepsis, with particularly high levels of expression of IL-6 and tumor necrosis factor (TNF), impaired acquired immunity (lymphopenia) and induced an uncontrolled innate response [31]. Interestingly, patients with viral infection such as influenza or COVID-19, in whom IPA has been reported at a high incidence, often present with low lymphocyte count. The high proportion of COVID-19 patients in our study may have favored lymphopenia. However, lymphopenia is common in ICU patients and frequently found to be a risk factor for secondary infections [32, 33]. Of note, beyond lymphopenia at ICU admission, persistent lymphopenia was found to be an important risk factor for ICU-acquired infection [33]. However, in the present study we could not assess whether persistent lymphopenia had a significant impact on IPA.

Strengths of our study include the multicentric design, systematic fungal culture of respiratory samples and score validation in a validation cohort. However, some limitations should be acknowledged. First, IPA diagnosis relies on clinical algorithms that include many specific measurements that are only performed if considered relevant by the clinician in charge. In the present study, only fungal dedicated pulmonary sample culture was systematically performed. As BAL and galactomannan screening were not systematically performed, IPA may also have been under-diagnosed. Nevertheless, by taking into account both possible and probable cases of IPA, we might have limited the underestimation of IPA cases. However, because the present study began during the first wave of SARS-COV 2 epidemic, when IPA was feared in critically ill COVID-19 patients [10], clinicians were aware of high incidence of IPA in mechanically ventilated patients and screening for fungal infection was common in participating ICUs which reduced selection bias. Secondly, patients were admitted in polyvalent ICUs with various reasons for admission making the population heterogeneous and possibly limiting the performance of the score developed in specific populations. In addition, almost all IPA patients were immunocompromised and/or admitted to the ICU with SARS-CoV2 infection, limiting the external validity of such a score. Therefore, external validity of the score in specific subpopulations should be evaluated and confirmation of our results on other cohorts will be necessary. Moreover, other un-reported confounders might be considered. Thirdly, although debated, the question of Aspergillus colonization versus infection could not be assessed in the present study. Thus, the inclusion of probable and possible IPA may have led to an overestimation of IPA. Nevertheless, recent findings suggest that a substantial proportion of Aspergillus cases (with a positive mycological criterion) in viral ARDS patients were in fact proven [34].

We constructed and validated an easily applicable PiPA score for prediction of IPA in patients with VAP which could be useful in identifying patients who might benefit from BAL with mycological screening. External validation will be needed to confirm our preliminary findings.

### Supplementary Information

**Additional file 1: Table S1**. Criteria for ICU acquired pulmonary aspergillosis. **Table S2**. Baseline characteristics and outcome in the learning and the validation cohorts. **Table S3**. Univariate analysis of predictor of invasive pulmonary aspergillosis. **Table S4**. Univariate analysis of predictor of invasive pulmonary aspergillosis in patients with or without immunodepression. **Figure S2**. Area under the operative curve in both cohorts. **Figure S3**. Survival curves (full population).

**Additional file 2: Figure 2**. Strategies for IPA diagnosis.

## Data Availability

The datasets generated during the current study are available from the corresponding author on reasonable request.
