# Supplementary material for: Prediction of pulmonary aspergillosis in patients with ventilator-associated pneumonia
Source: Ann Intensive Care. 2023 Nov 7;13:109. doi: 10.1186/s13613-023-01199-6 (PMC10630265; doi:10.1186/s13613-023-01199-6)
Supplement: Supplementary file 1 — Additional file 1: Table S1. Criteria for ICU acquired pulmonary aspergillosis. Table S2. Baseline characteristics and outcome in the learning and the validation cohorts. Table S3. Univariate analysis of predictor of invasive pulmonary aspergillosis. Table S4. Univariate analysis of predictor of invasive pulmonary aspergillosis in patients with or without immunodepression. Figure S2. Area under the operative curve in both cohorts. Figure S3. Survival curves (full population). [file 13613_2023_1199_MOESM1_ESM.docx]

**MATERIAL AND METHODS**

**Strategies for ICU acquired infection prevention**

As specified elsewhere, standard-care consists of semi recumbent position for all patients unless contra-indicated, cuff pressure monitoring every 6 hours and no specific protocol for stress ulcer prophylaxis. Notably there were no high-risk activities during study period in both ICUs, (no construction, no water leaks, windows were sealed and there were no plants in care units) (6). During the study period, multiple site decontamination was implemented in all participating ICUs (precise time of implementation is available in reference 12). Following implementation, all intubated patients with an expected mechanical ventilation duration > 24h received multiple site decontamination, consisting of administration of an antimicrobial suspension four times daily in the oropharynx and the gastric tube, chlorhexidine body-wash once daily and 5-days of nasal mupirocin throughout the duration of intubation. There was no systemic antibiotic treatment as part of this decontamination strategy (12).

**Statistical analysis**

Statistical analysis was performed with the statistical software R 4. 1. 1. Incidence rate and prevalence were expressed with a 95% confidence interval (95% CI), categorical variables as number (percentage), and continuous variables as median and interquartile range (IQR). When appropriate, the chi-square test and the Fisher exact test were used to compare categorical variables. The Man-Whitney U test and the Wilcoxon test were used for continuous variables when applicable. Multivariable logistic regression analysis was used to study risk factors. Multivariable logistic regression model was conducted with stepwise backward elimination using Akaike criteria as a stopping rule. For the purpose of score construction and in order to build an easily applicable score, relationships between continuous variables and IPA were analyzed by the receiver operator curve to define an optimal cut-off score conferring the best prediction values to discriminate patient at low versus high risk. For methodological issues regarding collinearity in between variables composing the “immunocompromised” status, the multivariable model was constructed with inclusion of the variable immunocompromised (yes/no) only without specifying ?the reason of immunodepression. All tests were two-sided, and a p value less than 0.05 was considered statistically significant. There were no missing data in this dataset.

**Table S1. Criteria for ICU acquired pulmonary aspergillosis.**

| Patients | Risk factors | Culture/PCR | Species | Antigenemia plasmatic/BAL | Chest tomography | Treatment |
| --- | --- | --- | --- | --- | --- | --- |
| 1 | HIV infection with acquired immunodeficiency syndrome | +/ NC | *NA* | 0.121/>6 | Alveolar opacity,  well-circumscribed  nodules | Voriconazole |
| 2 | Covid-19 | -/+ | *NA* | 0.107/2.2 | Alveolar opacity,  lobar consolidation | Voriconazole |
| 3 | Covid-19 | +/ NC | *Aspergillus fumigatus* | 0.174/- | Alveolar opacity,  well-circumscribed  nodules | Voriconazole |
| 4 | Dermatopolymyositis recquiring high dose steroids | -/+ | *Aspergillus fumigatus* | 0.122/>6 | Alveolar opacity,  lobar consolidation | Voriconazole |
| 5 | Melanoma, Pembrolizumab toxicity recquiring high dose steroids | +/ NA | *Aspergillus fumigatus* | 0.592/ NA | Lobar consolidation,  pleural effusion, cavitation | Voriconazole |
| 6 | Covid-19 | +/ NA | *Aspergillus fumigatus* | NA / NA | Diffuse reticular and alveolar opacity,  pleural effusion | Voriconazole |
| 7 | Covid-19 | +/ NA | *Aspergillus fumigatus* | NA / NA | Diffuse reticular and Alveolar opacity,  pleural effusion | Died before treatment |
| 8 | Covid-19 | +/+ | *Aspergillus fumigatus* | NA /1.88 | Lobar consolidation, pleural effusion, cavitation | Voriconazole |
| 9 | Covid-19 | +/ NA | *Aspergillus fumigatus* | 0.145/3.69 | Lobar consolidation, pleural effusion | Voriconazole |
| 10 | Covid-19  Renal transplantation | NA /+ | *NA* | 0.042/ NA | Alveolar opacity,  lobar consolidation, pleural effusion | Voriconazole |
| 11 | Covid-19 | +/ NA | *Aspergillus fumigatus* | 0.121/ NA | Diffuse reticular and alveolar opacity,  lobar consolidation | Voriconazole |
| 12 | Lung adenocarcinoma | +/ NA | *Aspergillus niger* | NA / NA | Alveolar opacity,  well-circumscribed  nodules | Died before treatment |
| 13 | Covid-19 | -/+ | *Aspergillus fumigatus* | 0.161/0.343 | Lobar consolidation, pleural effusion, cavitation, diffuse reticular and alveolar opacity | Voriconazole |
| 14 | Covid-19 | +/+ | *Aspergillus fumigatus* | NA / NA | Lobar consolidation, pleural effusion,  Alveolar opacity | Died before treatment |
| 15 | Influenza | +/+ | *Aspergillus fumigatus* | 0.346/ NA | Lobar consolidation, pleural effusion | Voriconazole |
| 16 | Chronic lymphoid leukemia | +/NA | *Aspergillus species* | Plasmatic BD glucane = 17 | NA | Voriconazole |
| 17 | Covid-19 | -/+ | *NA* | 0.06/4.6 | Bronchial wall thickening  lobar consolidation,  pleural effusion | Voriconazole |
| 18 | Covid-19 | +/NA | *Aspergillus fumigatus* | NA/>3 | Lobar consolidation | Died before treatment |
| 19 | Auto-immune encephalitis requiring high dose steroids | +/NA | *Aspergillus fumigatus* | NA/<0.2 | Diffuse reticular and Alveolar opacity, Bronchial wall thickening | Died before treatment |
| 20 | Alcoholic hepatitis requiring steroids | +/NA | *Aspergillus fumigatus* | NA/NA | Lobar consolidation | Died before treatment |
| 21 | Follicular lymphoma, Covid-19 | +/- | *Aspergillus fumigatus* | 0.45/NA | Diffuse reticular and alveolar opacity | Died before treatment |
| 22 | Varicelle-zona infection | +/+ | *Aspergillus fumigatus* | 0.0/1.2 | Lobar consolidation, pleural effusion | Voriconazole |
| 23 | Chronic lymphoid leukemia | +/NA | *Aspergillus fumigatus* | 0.04/NA | Bronchial wall thickening, nodular consolidation | Voriconazole |
| 24 | Covid-19 | +/NA | *Aspergillus fumigatus* | 0.12/>6.0 | Diffuse reticular and alveolar opacity,  pleural effusion | Voriconazole |
| 25 | Covid-19 | +/NA | *Aspergillus fumigatus* | NA/NA | Diffuse reticular and alveolar opacity, Bronchial wall thickening,  pleural effusion | Diminution of steroids and immunomodulatory treatment cessation |
| 26 | NA | +/+ | *Aspergillus fumigatus* | 0.118/>9.0 | Alveolar opacity, nodular consolidation | NA |
| 27 | Covid-19 | +/+ | *Aspergillus fumigatus* | NA/NA | Alveolar opacity,, nodular consolidation | NA |
| 28 | Lung adenocarcinoma | +/+ | *Aspergillus fumigatus* | NA/>6.0 | Bronchial wall thickening,, nodular consolidation | NA |
| 29 | Diffuse Large B cell Lyphoma | +/NA | *Aspergillus fumigatus* | 0.118/NA | Diffuse reticular and Aaveolar opacity,  pleural effusion | Died before treatment |
| 30 | Covid-19  Lung adenocarcinoma | -/+ | *NA* | 0.77/NA | Diffuse reticular and alveolar opacity | Voriconazole |
| 31 | Covid-19 | +/NA | *Aspergillus fumigatus* | 0.07/NA | Diffuse reticular and alveolar opacity | Voriconazole |
| 32 | Covid-19 | -/+ | *NA* | NA/NA | Diffuse reticular and alveolar opacity | Voriconazole |
| 33 | Covid-19 | +/NA | *Aspergillus fumigatus* | 0.07/1.2 | Diffuse reticular and alveolar opacity | Voriconazole |
| 34 | Covid-19  Multiple myeloma | +/NA | *Aspergillus fumigatus* | 0.04/NA | NA | Voriconazole |
| 35 | Covid-19 | +/NA | *Aspergillus fumigatus* | 0.0/NA | NA | Voriconazole |
| 36 | Covid-19 | +/NA | *Aspergillus fumigatus* | NA/NA | Diffuse reticular and alveolar opacity, nodular consolidation | Voriconazole |
| 37 | Covid-19 | +/NA | *Aspergillus fumigatus* | 0.0/>1.0 | NA | Voriconazole |
| 38 | Covid-19 | +/NA | *Aspergillus fumigatus* | NA/>1.0 | Diffuse reticular and alveolar opacity,  pleural effusion | Voriconazole |
| 39 | Renal transplantation | +/NA | *Aspergillus fumigatus* | >0.5/NA | Alveolar opacity | Voriconazole |

**Table S2. Baseline characteristics and outcome in the learning and the validation cohorts.**

|  | Validation cohort | Learning cohort | p-value |
| --- | --- | --- | --- |
| Variables | n = 105 | n = 110 |  |
| Age, year | 65 [53 - 72] | 64 [58 - 70] | 0.965 |
| Male – no°. (%) | 81 (77.1) | 88 (80.0) | 0.731 |
| Year of admission |  |  | 0.993 |
| 2020 – no. (%) | 38 (36.2) | 39 (35.5) |  |
| 2021 – no. (%) | 47 (44.8) | 50 (45.5) |  |
| 2022 – no. (%) | 20 (19.0) | 21 (19.1) |  |
| *Immunocompromised – no. (%)* | 31 (29.5) | 28 (25.5) | 0.606 |
| Oncohematological disorder – no. (%) | 20 (19.0) | 18 (16.4) | 0.721 |
| Immunomodulatory treatment – no. (%) | 14 (14.0) | 9 (9.4) | 0.433 |
| Solid organ transplant – no. (%) | 3 (2.9) | 3 (2.7) | 1.000 |
| Neutropenia <0.5 G/L – no. (%) | 3 (2.9) | 9 (8.2) | 0.161 |
| Steroids | 55 (52.4) | 51 (46.4) | 0.456 |
| < 1mg/kg of prednisolone equivalent | 45 (46.9) | 41 (41.8) | 0.574 |
| ≥ 1mg/kg of prednisolone equivalent | 10 (9.5) | 12 (10.9) | 0.824 |
| Simplified acute physiology score II | 50 [38 - 61] | 48 [37 - 68] | 0.828 |
| *Reason for admission* |  |  |  |
| Medical (vs surgical) – no. (%) | 50 [38 - 61] | 48 [37 - 68] | 0.828 |
| Trauma – no. (%) | 4 (3.8) | 8 (7.3) | 0.375 |
| COVID-19 – no. (%) | 63 (60.0) | 59 (53.6) | 0.422 |
| Influenza – no. (%) | 2 (2.3) | 1 (1.2) | 1.000 |
| *Period of admission* |  |  | 0.138 |
| Winter – no. (%) | 30 (28.6) | 36 (32.7) |  |
| Spring – no. (%) | 36 (34.3) | 24 (21.8) |  |
| Summer – no. (%) | 11 (10.5) | 20 (18.2) |  |
| Fall – no. (%) | 28 (26.7) | 30 (27.3) |  |
| *Localization before admission* |  |  | 0.301 |
| Other ICU – no. (%) | 10 (9.5) | 20 (18.2) |  |
| Home – no. (%) | 51 (48.6) | 52 (47.3) |  |
| Long care facility – no. (%) | 2 (1.9) | 2 (1.8) |  |
| Acute care ward – no. (%) | 42 (40.0) | 36 (32.7) |  |
| *Early management* |  |  |  |
| Systemic antibiotic at admission – no. (%) | 67 (63.8) | 78 (70.9) | 0.335 |
| Vascular catheter – no. (%) | 100 (95.2) | 108 (98.2) | 0.406 |
| Multiple site decontamination – no. (%) | 26 (24.8) | 33 (30.0) | 0.479 |
| *Biological parameters at admission* |  |  |  |
| Creatininemia, µmol/L | 84 [62.75 - 150] | 85 [64 - 132.50] | 0.882 |
| White blood cell count, Giga/L | 10.40 [7.20 - 16.40] | 10.73 [8.56 - 15.65] | 0.954 |
| Lymphocyte, Giga/L | 0.70 [0.44 - 1.20] | 0.76 [0.51 - 1.27] | 0.426 |
| PaO2/FiO2, mmHg | 127.50 [90 - 175] | 137 [100.75 - 208.50] | 0.135 |
| *Outcomes* |  |  |  |
| In ICU death – no. (%) | 43 (41.0) | 30 (27.3) | 0.048 |
| Length of stay, days | 25 [16 - 36.25] | 28 [18 - 44.50] | 0.276 |
| Length of mechanical ventilation, days | 21 [12 - 33] | 22.50 [13 - 37] | 0.633 |
| ICU acquired pulmonary aspergillosis – no. (%) | 23 (21.9) | 16 (14.5) | 0.215 |
| Putative/possible – no. (%) | 0 (0.0) | 4 (4.4) | 0.121 |
| Probable – no. (%) | 23 (25.8) | 12 (13.3) | 0.039 |

Note. SC : standard care. MSD : Multiple site decontamination. SMD : Standard mean difference. ICU : Intensive-care unit. COVID-19 : SARS-COV 2 associated infection disease. *> 1 mg/kg of equivalent prednisolone.

**Table S3. Univariate analysis of predictor of invasive pulmonary aspergillosis.**

|  | Full population | |  | Patients with BAL for VAP diagnosis  (n = 123) | |  |
| --- | --- | --- | --- | --- | --- | --- |
| Variables | OR | 95% CI | p-value | OR | 95% CI | p-value |
| Age, per supplementary year | 1.04 | 0.01-1.08 | 0.007 | 1.05 | 1.01-1.09 | 0.012 |
| Male | 1.62 | 0.63-4.14 | 0.32 | 1.5 | 0.54-4.14 | 0.433 |
| Year of admission, per supplementary year | 1.23 | 0.77-1.99 | 0.39 | 0.74 | 0.45-1.23 | 0.243 |
| *Immunocompromised** | 4.86 | 2.34-10.08 | <0.001 | 4.00 | 1.80-8.90 | <0.001 |
| Oncohematological disorder | 2.56 | 1.15-5.69 | 0.021 | 2.43 | 0.99-5.98 | 0.054 |
| Immunomodulatory treatment | 9.37 | 3.39-22.79 | <0.001 | 5.94 | 2.24-15.71 | <0.001 |
| Solid organ transplant | 4.80 | 0.93-24.78 | 0.061 | 2.25 | 0.43-11.69 | 0.335 |
| Neutropenia <0.5 G/L | 7.48 | 2.23-25.04 | 0.001 | 4.37 | 1.20-15.98 | 0.025 |
| Steroids | 3.73 | 1.71-8.12 | <0.001 | 3.51 | 1.52-8.11 | 0.003 |
| < 1 mg/kg of equivalent prednisolone | 2.42 | 1.04-5.59 | 0.039 | 2.25 | 0.93-5.42 | 0.071 |
| ≥ 1 mg/kg of equivalent prednisolone | 9.28 | 3.61-23.87 | <0.001 | 13.5 | 3.57-51.09 | <0.001 |
| Simplified acute physiology score II, per 1 point increment | 0.98 | 0.96-1.00 | 0.13 | 0.99 | 0.96-1.01 | 0.211 |
| *Reason for admission* |  |  |  |  |  |  |
| Medical (vs surgical) | inf | 0-inf | 0.99 | inf | 0-inf | 0.99 |
| Trauma | 0 | 0-inf | 0.99 | 0 | 0-inf | 0.99 |
| Viral infection | 1.83 | 0.87-3.84 | 0.11 | 1.85 | 0.83-4.15 | 0.13 |
| COVID-19 | 1.45 | 0.71-2.98 | 0.31 | 1.47 | 0.67-3.23 | 0.330 |
| Influenza | 10.30 | 0.90-117.62 | 0.061 | 5.18 | 0.45-59.56 | 0.186 |
| *Period of admission* |  |  |  |  |  |  |
| Winter | 0.85 | 0.35-2.08 | 0.72 | 0.76 | 0.28-2.06 | 0.590 |
| Spring | 0.59 | 0.22-1.57 | 0.29 | 0.47 | 0.16-1.37 | 0.166 |
| Summer | 1.12 | 0.39-3.21 | 0.72 | 0.85 | 0.26-2.74 | 0.789 |
| Fall | Ref | Ref | Ref | Ref | Ref | Ref |
| *Localization before admission* |  |  |  |  |  |  |
| Other ICU | 0.12 | 0.02-0.98 | 0.047 | 0.11 | 0.01-0.88 | 0.037 |
| Home | 0.92 | 0.45-1.89 | 0.81 | 1.13 | 0.50-2.52 | 0.772 |
| Acute care ward | Ref | Ref | Ref | Ref | Ref | Ref |
| *Early management* |  |  |  |  |  |  |
| Systemic antibiotic at admission | 1.77 | 0.79-3.96 | 0.17 | 1.26 | 0.52-3.05 | 0.613 |
| Vascular catheter | inf | 0-inf | 0.99 | inf | 0-inf | 0.99 |
| Multiple-site decontamination | 1.63 | 0.78-3.41 | 0.19 | 0.74 | 0.34-1.64 | 0.465 |
| *Biological parameters at admission* |  |  |  |  |  |  |
| Creatininemia, per supplementary µmol/L | 0.99 | 0.99-1.00 | 0.50 | 1.00 | 0.99-1.00 | 0.601 |
| White blood cell count, per supplementary Giga/L | 0.94 | 0.88-1.00 | 0.056 | 0.96 | 0.90-1.02 | 0.160 |
| Lymphocyte, per supplementary Giga/L | 0.22 | 0.09-0.55 | 0.001 | 0.24 | 0.10-0.63 | 0.003 |
| PaO2/FiO2, per supplementary mmHg | 0.99 | 0.98-0.99 | 0.008 | 0.99 | 0.99-1.00 | 0.113 |
| *Characteristics at ventilator-associated pneumonia onset* |  |  |  |  |  |  |
| Temperature, per supplementary °C | 0.64 | 0.40-1.03 | 0.066 | 0.91 | 0.56-1.47 | 0.694 |
| White blood cell count, per supplementary Giga/L | 0.91 | 0.83-0.99 | 0.025 | 0.91 | 0.83-0.99 | 0.032 |
| PaO2/FiO2, per supplementary mmHg | 0.99 | 0.99-1.00 | 0.076 | 1.00 | 0.99-1.01 | 0.844 |
| Shock | 0.67 | 0.29-1.54 | 0.35 | 0.82 | 0.33-2.03 | 0.665 |
| Purulent sputum | 0.23 | 0.09-0.58 | 0.001 | 0.38 | 0.145-0.989 | 0.047 |
| Time since admission , per supplementary days | 1.00 | 0.96-1.04 | 0.85 | 0.97 | 0.93-1.01 | 0.199 |

* For methodological issues regarding collinearity in between variables composing the “immunocompromised” status, the multivariable model was constructed with inclusion of the variable immunocompromised (yes/no) only without specifying the reason of immunodepression.

**Table S4. Univariate analysis of predictor of invasive pulmonary aspergillosis in patients with or without immunodepression.**

|  | Patients with preexisting  immunodepression$ (n = 136) | |  | Immunocompetent patients  (n = 79) | |  |
| --- | --- | --- | --- | --- | --- | --- |
| Variables | OR | 95% CI | p-value | OR | 95% CI | p-value |
| Age, per supplementary year | 1.05 | 1.00-1.10 | 0.030 | 1.04 | 0.99-1.08 | 0.110 |
| Male | 1.90 | 0.60-5.99 | 0.27 | 2.17 | 0.16-10.11 | 0.325 |
| Year of admission, per supplementary year | 0.95 | 0.54-1.66 | 0.86 | 0.88 | 0.41-1.90 | 0.746 |
| *Immunocompromised** | 4.60 | 1.85-11.44 | 0.001 | - | - | - |
| Oncohematological disorder | 2.36 | 0.99-5.64 | 0.052 | - | - | - |
| Immunomodulatory treatment | 16.82 | 4.80-58.95 | <0.001 | - | - | - |
| Solid organ transplant | 4.20 | 0.80-22.06 | 0.090 | - | - | - |
| Neutropenia <0.5 G/L | 6.87 | 1.99-23.73 | 0.002 | - | - | - |
| Steroids | 2.76 | 0.89-8.57 | 0.079 | - | - | - |
| < 1 mg/kg of equivalent prednisolone | 2.56 | 0.70-9.34 | 0.154 | - | - | - |
| ≥ 1 mg/kg of equivalent prednisolone | 6.11 | 2.17-17.14 | <0.001 | - | - | - |
| Simplified acute physiology score II, per 1 point increment | 0.99 | 0.97-1.02 | 0.69 | 0.95 | 0.92-0.99 | 0.024 |
| *Reason for admission* |  |  |  |  |  |  |
| Medical (vs surgical) | inf | 0-inf | 0.99 | inf | 0-inf | 0.990 |
| Trauma | 0 | 0-inf | 0.99 | 0 | 0-inf | 0.991 |
| Viral infection | 0.91 | 0.39-2.13 | 0.83 | inf | 0-inf | 0.990 |
| COVID-19 | 0.87 | 0.37-2.05 | 0.76 | 5.93 | 1.29-27.34 | 0.022 |
| Influenza |  |  |  | 25.75 | 2.10-315.58 | 0.011 |
| *Period of admission* |  |  |  |  |  |  |
| Winter | 0.97 | 0.32-2.90 | 0.95 | 1.01 | 0.28-3.62 | 0.987 |
| Spring | 0.62 | 0.18-2.12 | 0.45 | 0.31 | 0.06-1.71 | 0.180 |
| Summer | 1.94 | 0.59-6.40 | 0.28 | 0.61 | 0.11-3.43 | 0.575 |
| Fall | Ref | Ref | Ref | ref | ref | Ref |
| *Localization before admission* |  |  |  |  |  |  |
| Other ICU | 0.29 | 0.03-2.42 | 0.25 | 0 | 0-inf | 0.991 |
| Home | 2.08 | 0.86-5.05 | 0.105 | 0.62 | 0.21-1.84 | 0.395 |
| Acute care ward | Ref | Ref | Ref | ref | ref | Ref |
| *Early management* |  |  |  |  |  |  |
| Systemic antibiotic at admission | 1.38 | 0.53-3.55 | 0.51 | 2.51 | 0.68-9.35 | 0.168 |
| Vascular catheter | inf | 0-inf | 0.99 | inf | 0-inf | 0.990 |
| Multiple-site decontamination | 1.31 | 0.57-3.02 | 0.53 | 0.85 | 0.22-3.21 | 0.811 |
| *Biological parameters at admission* |  |  |  |  |  |  |
| Creatininemia, per supplementary µmol/L | 0.99 | 0.99-1.00 | 0.73 | 0.99 | 0.98-1.00 | 0.171 |
| White blood cell count, per supplementary Giga/L | 0.98 | 0.92-1.05 | 0.60 | 0.91 | 0.81-1.02 | 0.118 |
| Lymphocyte, per supplementary Giga/L | 0.24 | 0.07-0.78 | 0.018 | 0.31 | 0.08-1.22 | 0.094 |
| PaO2/FiO2, per supplementary mmHg | 0.99 | 0.99-1.00 | 0.29 | 0.98 | 0.97-0.99 | 0.017 |
| *Characteristics at ventilator-associated pneumonia onset* |  |  |  |  |  |  |
| Temperature, per supplementary °C | 0.62 | 0.36-1.05 | 0.074 | 1.04 | 0.50-2.15 | 0.922 |
| White blood cell count, per supplementary Giga/L | 0.91 | 0.82-1.00 | 0.054 | 0.91 | 0.79-1.05 | 0.215 |
| PaO2/FiO2, per supplementary mmHg | 1.00 | 0.99-1.01 | 0.39 | 0.99 | 0.98-1.00 | 0.173 |
| Shock | 0.59 | 0.22-1.63 | 0.31 | 0.32 | 0.07-1.52 | 0.154 |
| Purulent sputum | 0.31 | 0.09-1.04 | 0.058 | 0.15 | 0.04-0.51 | 0.003 |
| Time since admission , per supplementary days | 1.00 | 0.96-1.04 | 0.88 | 1.02 | 0.97-1.07 | 0.462 |

^$^ Prexisting immunodepression corresponds to onco-hematological disorder, immunomodulatory treatment when administered before admission, solid organ transplant, neutropenia < 0.5G/L when present before admission.

* For methodological issues regarding collinearity in between variables composing the “immunocompromised” status, the multivariable model was constructed with inclusion of the variable immunocompromised (yes/no) only without specifying the reason of immunodepression.


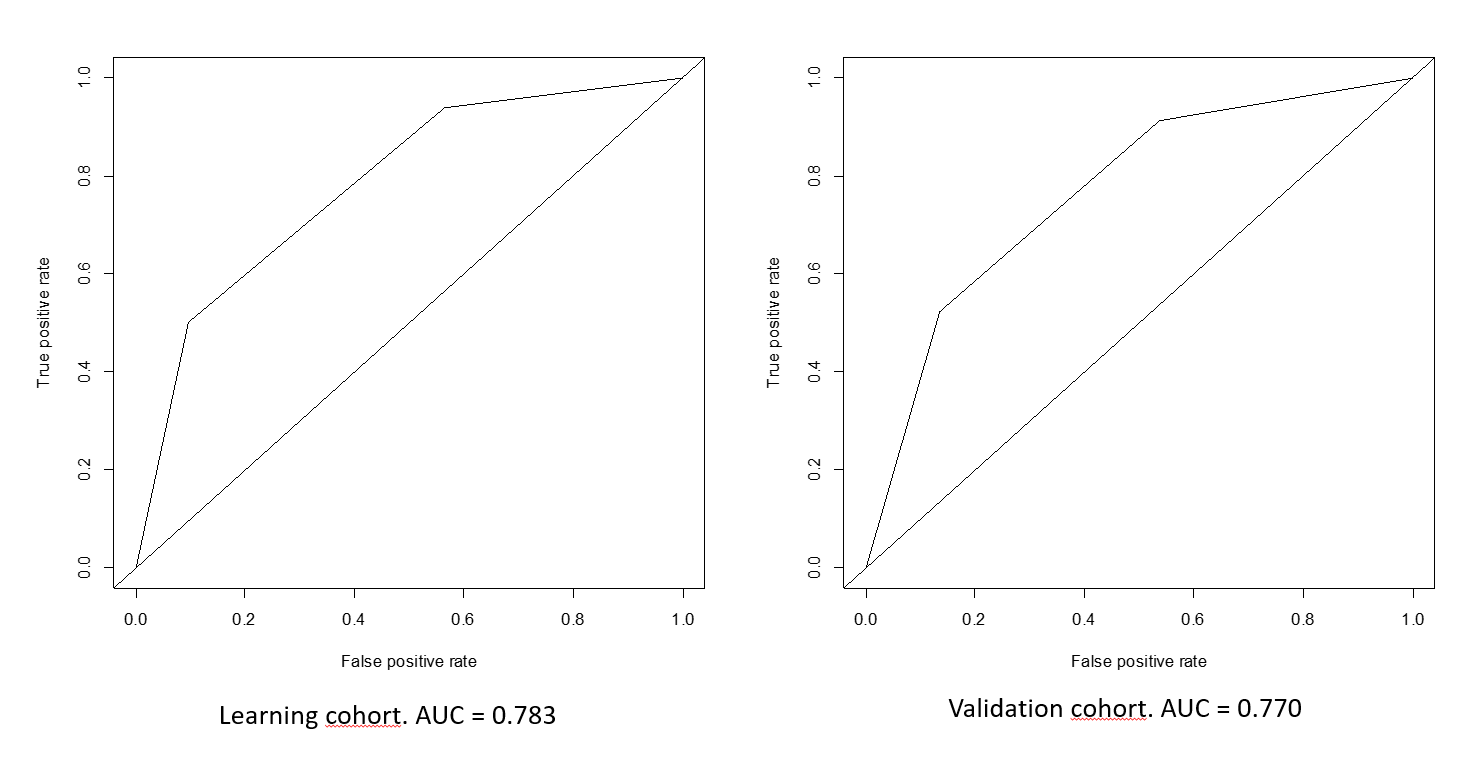


**Figure S2. Area under the operative curve in both cohorts.**

**Figure S3. Survival curves (full population).**
