# Supplementary material for: Prediction of pulmonary aspergillosis in patients with ventilator-associated pneumonia
Source: Ann Intensive Care. 2023 Nov 7;13:109. doi: 10.1186/s13613-023-01199-6 (PMC10630265; doi:10.1186/s13613-023-01199-6)
Supplement: Supplementary file 2 — Additional file 2: Figure 2. Strategies for IPA diagnosis. [file 13613_2023_1199_MOESM2_ESM.pptx]

## Slide 1
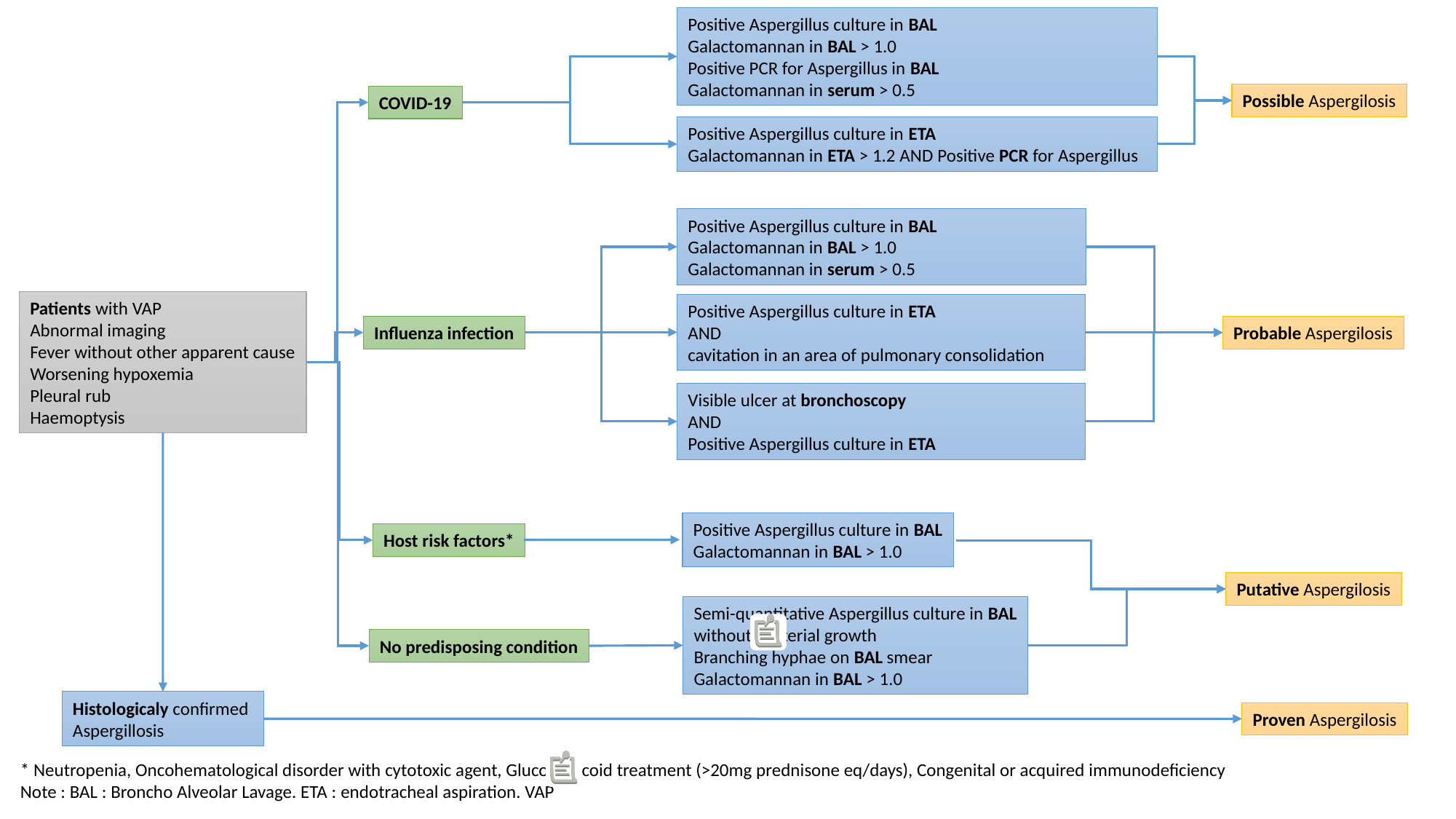

Positive Aspergillus culture in BAL
Galactomannan in BAL > 1.0
Positive PCR for Aspergillus in BAL
Galactomannan in serum > 0.5
Possible Aspergilosis
COVID-19
Positive Aspergillus culture in ETA
Galactomannan in ETA > 1.2 AND Positive PCR for Aspergillus
Positive Aspergillus culture in BAL
Galactomannan in BAL > 1.0
Galactomannan in serum > 0.5
Patients with VAP
Abnormal imaging
Fever without other apparent cause
Worsening hypoxemia
Pleural rub
Haemoptysis
Positive Aspergillus culture in ETA
AND
cavitation in an area of pulmonary consolidation
Influenza infection
Probable Aspergilosis
Visible ulcer at bronchoscopy
AND
Positive Aspergillus culture in ETA
Positive Aspergillus culture in BAL
Galactomannan in BAL > 1.0
Host risk factors*
Putative Aspergilosis
Semi-quantitative Aspergillus culture in BAL
without bacterial growth
Branching hyphae on BAL smear
Galactomannan in BAL > 1.0
No predisposing condition
Histologicaly confirmed
Aspergillosis
Proven Aspergilosis
* Neutropenia, Oncohematological disorder with cytotoxic agent, Glucocorticoid treatment (>20mg prednisone eq/days), Congenital or acquired immunodeficiency
Note : BAL : Broncho Alveolar Lavage. ETA : endotracheal aspiration. VAP
